# Prevalence of refractive errors among school-going children in a multistate study in India

**DOI:** 10.1136/bjo-2022-322123

**Published:** 2022-12-05

**Authors:** Elizabeth Joseph, Meena CK, Rahul Kumar, Mary Sebastian, Catherine M Suttle, Nathan Congdon, Sheeladevi Sethu, Gudlavalleti VS Murthy

**Affiliations:** 1 Department of Ophthalmology, Little Flower Hospital and Research Centre, Angamaly, Kerala, India; 2 Orbis India Country office, ORBIS International, Gurugram, Haryana, India; 3 Optometry and Visual Science, City University London, London, UK; 4 Research, Orbis International, New York, New York, USA; 5 Indian Institute of Public Health, Hyderabad, India

**Keywords:** Optics and Refraction, Epidemiology

## Abstract

**Aim:**

Much existing data on childhood refractive error prevalence in India were gathered in local studies, many now dated. The aim of this study was to estimate the prevalence, severity and determinants of refractive errors among school-going children participating in a multistate vision screening programme across India.

**Methods:**

In this cross-sectional study, vision screening was conducted in children aged 5–18 years at schools in five states using a pocket vision screener. Refractive error was measured using retinoscopy, and subjective refraction and was defined both by spherical equivalent (SE) and spherical ametropia, as myopia ≤−0.5 diopters (D), hyperopia ≥+1.0 D and/or astigmatism as >0.5 D. Data from the eye with less refractive error were used to determine prevalence.

**Results:**

Among 2 240 804 children (50.9% boys, mean age 11.5 years, SD ±3.3), the prevalence of SE myopia was 1.57% (95% CI 1.54% to 1.60%) at 5–9 years, 3.13% (95% CI 3.09% to 3.16%) at 10–14 years and 4.8% (95% CI 4.73% to 4.86%) at 15–18 years. Hyperopia prevalence was 0.59% (95% CI 0.57% to 0.61%), 0.54% (95% CI 0.53% to 0.56%) and 0.39% (95% CI 0.37% to 0.41%), respectively. When defined by spherical ametropia, these values for myopia were 0.84%, 2.50% and 4.24%, and those for hyperopia were 2.11%, 2.41% and 2.07%, respectively.

Myopia was associated with older age, female gender, private school attendance, urban location and state. The latter appeared to be driven by higher literacy rates.

**Conclusions:**

Refractive error, especially myopia, is common in India. Differences in prevalence between states appear to be driven by literacy rates, suggesting that the burden of myopia may rise as literacy increases.

**WHAT IS ALREADY KNOWN ON THIS TOPIC**

- Prevalence of refractive error among school children has been determined within regions in India.

**WHAT THIS STUDY ADDS**

- This study determines and compares refractive error in children within and between states, rural and urban regions, and attending government-funded and privately funded schools in India.

**HOW THIS STUDY MIGHT AFFECT RESEARCH, PRACTICE OR POLICY**

- The results show that refractive error prevalence among school children may increase with literacy rates in India and highlight a need for strategies to address this potential issue.

## Introduction

Visual impairment in childhood has a negative impact on socioemotional competence and academic development.^1^ Uncorrected refractive error (URE) is the leading cause of vision impairment and the second leading cause of blindness globally,^2^ affecting 1 in 90 people of all ages.^3^ Available evidence indicates that URE in school-aged children continues to be a major public health problem in India.^4^

The impact of URE in children depends on a range of factors, including the type (myopia or hyperopia), severity and working distance for different tasks. Uncorrected hyperopia may result in accommodation-related and strabismus-related impact on quality of life,^5^ attention and learning.^6^ Uncorrected myopia may have a negative impact on distance tasks such as viewing a blackboard, impacting not only educational outcomes^7^ but also self-esteem and well-being.^8^ In addition, uncorrected anisometropia in early childhood may result in amblyopia, with associated loss of depth perception, impacting activities of daily living.^9^ Early correction of childhood refractive error is therefore important to prevent these adverse effects.

At birth, the eye is normally mildly hyperopic, and this error reduces over the next several years. The risk of myopia in childhood is associated with a range of socioenvironmental factors, with indoor lifestyle and with more time on schooling and other near-work tasks.^10 11^ Myopia is associated with urban locations,^10^ but near tasks related to mobile phones, tablets and other technologies are common and influence refractive error in both rural and urban locations.

Most existing population-based data on the prevalence of refractive error among Indian children were gathered in local rather than multisite studies.^4 12 13^ Children’s access to school education in India has improved in recent years^14^ and may have increased myopia prevalence. In view of the impact of URE on childhood development, updated and nationally representative prevalence data on children’s refractive error in India are needed.

Most prior studies of refractive error prevalence among children in India have reported spherical equivalent (SE), the algebraic sum of the spherical and half of the cylindrical component. While astigmatism is often also reported, SE measures reduce hyperopic and increase myopic errors when cylinder power is negative. This may exclude some hyperopic children from prevalence data and increase the apparent prevalence of myopia. For this reason, in the present study, refractive error prevalence was estimated using both SE and spherical ametropia without adjustment for the cylinder. Using these two approaches, the study reports the prevalence of refractive errors and their determinants among school-going children participating in a large, multistate vision screening and spectacle delivery programme in India.

## Methods

### Study population

This prevalence study was conducted as part of Orbis International’s Refractive Errors Among Children (REACH) school vision screening and eye care service delivery programme.^15 16^ Data were collected between July 2016 and December 2019. In the area served by each partner hospital, one revenue district not previously covered by school vision screening was selected. Within each district, all schools willing to participate were included. All children from grades 1 to 12 in the selected schools were eligible for inclusion.

### Refractive error screening procedure

A team consisting of a project officer, project coordinator, trained primary vision screeners, optometrist or vision technician, counsellor and optician visited each school. Data relating to each student participant including age, gender, grade and the details of the coordinating teacher were obtained in advance from school authorities and entered into the REACH custom software (REACHSoft). REACHSoft is a paperless system designed to store data relating to each school and student, including basic and detailed ocular evaluations, refractive power, spectacle dispensing and compliance with spectacle wear. REACHSoft also facilitates progress monitoring, report generation and data management in the field, without reliance on internet access.

The date of screening was established well in advance and was provided to parents through the school administration to minimise the number of absentees. All participants present at school underwent primary screening conducted by trained project staff. This included basic vision and eye screening using a pocket vision screener^17^ to present optotypes (letters or numbers) at a standard size and distance consistent with a Snellen visual acuity of 6/9.5 (logarithm of the minimal angle of resolution (logMAR) 0.2) to detect visually significant myopia and/or astigmatism. A +1.50 diopter (D) spectacle test was used to relax accommodation and thus reveal any hyperopia masked by accommodation (latent hyperopia). These tests were followed by a torch light eye examination to look for external ocular abnormalities, including media opacities, pupillary defects and strabismus. The screening was conducted in a classroom which allowed a viewing distance of 3 m and with moderate ambient room illumination to enable visual acuity testing. Additional equipment including a 3 m measuring tape (to set the appropriate viewing distance), a torch, occluders (for monocular testing) and a power bank to charge the tablet were made available to the screening team. Monocular vision testing was carried out with and without the +1.50 D spectacles, starting with the right eye.

Children unable to read the 0.2 logMAR optotypes monocularly unaided, or only able to read them with the aid of a +1.50 D lens, those already wearing prescription spectacles and those with a complaint such as headache, lacrimation or discomfort were invited to undergo a detailed ocular evaluation by a qualified optometrist or a trained vision technician.

Objective refraction was conducted using a streak retinoscope and occasionally with a portable, handheld vision screening device (Welch Allen, Skaneateles Falls, New York, USA) before monocular subjective refraction was conducted using a trial frame and retroilluminated logMAR charts to measure best-corrected visual acuity. To check the accuracy of non-cycloplegic retinoscopy, accommodative lag was measured using the monocular estimation method of dynamic retinoscopy. Cycloplegic retinoscopy was performed on children aged 10 years or less or with any of the following in either eye:

- Refractive error associated with esodeviation.
- Hyperopia with asthenopic symptoms (such as headache and ocular discomfort).
- >2 D difference between existing spectacle correction and the correction determined during detailed evaluation.
- Fluctuating retinoscopy readings.
- Myopia with >0.75 D discrepancy between retinoscopy and the subjective result.
- Dynamic retinoscopy lag >1.00 D.

Torch light examination was repeated to assess the anterior segment. All children whose vision in either eye did not improve beyond logMAR 0.2 with refraction, or in whom the cause of visual impairment could not be determined, were referred to the nearest base hospital.

### Definition of refractive error

Spherical refractive error was recorded in two different ways: (1) as SE (the algebraic sum of spherical and 0.5× cylindrical component) and (2) as the spherical component without adjustment. For example, a refractive error of +1.50/–1.00 ×90 would be recorded as (1) +1.00 and (2) +1.50. Myopia was defined as ≤−0.5 D SE,^18^ hyperopia as ≥+1.0 D SE and astigmatism as ≥−0.50 D. Astigmatism was expressed as simple myopic (zero spherical error with negative cylinder), simple compound (negative spherical and cylindrical errors) and mixed astigmatism (positive spherical error and negative cylinder). Anisometropia was defined as an interocular SE difference of >1.0 D. Prevalence of refractive error was calculated as the sum of myopia and hyperopia as defined previously using data from both primary screening and detailed evaluation.

### Statistical analyses

Data from the eye with lower refractive error (referred to as the better eye in this paper) were used to calculate prevalence of refractive error. Distributions of refractive errors according to gender, state and other factors were based on data from the better eye (with lower SE). Mean±SD is reported for continuous variables with normal distributions and median±first and third quartiles for those with non-normal distributions. Frequencies are reported for categorical variables. The proportions of myopia, hyperopia, astigmatism and anisometropia and their 95% CIs were calculated within groups based on age, gender, state, urban and rural locations.

Population size of the area in which each school was located was used to classify each school as rural (population under 5000), semirural (5000–99 999), semiurban (100 000–999 999) or urban (over 1 000 000). These definitions are in accordance with those used by the Census of India.^19^

Univariate and multivariate logistic regression analyses were conducted to assess factors associated with the presence of myopia (SE ≤−0.50 better eye). Factors considered in regression analyses were age, gender, urban or rural location and population size. The OR and corresponding 95% CIs were calculated to identify myopia risk factors. In the model, OR of >1.0 and p value of <0.05 indicated a risk factor, while OR of <1.0 and p value of <0.05 indicated a protective factor. All statistical tests were two-sided, and a p value of <0.05 was considered statistically significant. All statistical analyses were performed using Stata V.14.

## Results

A total of 2 240 804 children in 10 309 schools participated in this study. Most (78.7%) were in rural locations and over three-quarters (75.9%) were government-aided (receiving public funding). Almost all of the children completed primary screening: 90.3% overall; 89.7% and 90.8%, respectively, in urban and rural locations; 89.4% vs 91.4%, respectively, in government and private schools. A total of 174 706 (8.6%) children were referred for detailed evaluation and 137 148 (78.5%) of them completed this process. The proportions of boys and girls who were enrolled in the study underwent primary screening, were identified for and completed detailed evaluation were similar (table 1).

**Table 1:** Distribution of refractive errors (better eye) by age and gender

Boys	Age (years)				P value comparing different ages
	5–9 (n=12 470)	10–14 (n=28 377)	15–18 (n=13 985)	Total (n=54 832)	
SE (D)	n (%)				
<−6.0	155 (1.24)	371 (1.31)	208 (1.49)	734 (1.34)	<0.001
<−3.0 to −6.0	308 (2.47)	1455 (5.13)	1133 (8.10)	2896 (5.28)	
<−0.5 to −3.0	3866 (31.00)	10 564 (37.23)	6231 (44.55)	20 661 (37.68)	
≥−0.5 to ≤+0.5	5328 (42.73)	12 223 (43.07)	5288 (37.81)	22 839 (41.65)	
>0.5 to 2.0	1873 (15.02)	2584 (9.11)	866 (6.19)	5323 (9.71)	
>2.0	940 (7.54)	1180 (4.16)	259 (1.85)	2379 (4.34)	
Median refractive error (D) (first and third quartiles)	−0.25 (−0.88, 0.25)	−0.50 (−1.25, 0.00)	−0.75 (–1.63, 0.25)	−0.50 (–1.5, 0.50)	<0.001
Range	−18 to 13.0	−20.5 to 16.0	−20.75 to 19.0	−20.75 to 19.0	
Median cylinder (D) (first and third quartiles)	−1.0 (–1.75, –0.50)	−0.75 (–1.25, –0.50)	−0.75 (–1.00, –0.50)	−0.75 (–1.50, –0.50)	<0.001
Range	−7.75 to 0	−7.75 to 0	−7.75 to 0	−7.75 to 0	

Girls	5–9 (n=11 014)	10–14 (n=31 767)	15–18 (n=19 103)	Total (n=61 884)	
SE (D)	n (%)				
<−6.0	114 (1.04)	382 (1.20)	280 (1.47)	776 (1.25)	<0.001
<−3.0 to −6.0	255 (2.32)	1822 (5.74)	1723 (9.02)	3800 (6.14)	
<−0.5 to −3.0	3429 (31.13)	11 662 (36.71)	8244 (43.16)	23 335 (37.71)	
≥−0.5 to ≤+0.5	4839 (43.93)	14 232 (44.80)	7590 (39.73)	26 661 (43.08)	
>0.5 to 2.0	1647 (14.95)	2700 (8.50)	973 (5.09)	5320 (8.60)	
>2.0	730 (6.63)	969 (3.05)	293 (1.53)	1992 (3.22)	
Median refractive error (D) (first and third quartiles)	−0.25 (−0.86, 0.25)	−0.50 (−1.25, 0.00)	−0.75 (−1.75, –0.25)	−0.5 (−1.25, –0.50)	<0.001
Range	−21.75 to 18.86	−21.75 to 13.0	−20.75 to 15.25	−21.75 to 18.86	
Median cylinder (D) (first and third quartiles)	−1.0 (−1.50, –0.50)	−0.75 (−1.00, –0.50)	−0.5 (−1.00, –0.50)	−0.75 (−1.25, –0.50)	<0.001
Range	−7.75 to 0	−7.75 to 0	−6.5 to 0	−7.75 to 0	

Overall	5–9 (n=23 484)	10–14 (n=60 144)	15–18 (n=33 088)	Total (n=116 716)	
SE (D)	n (%)				
<−6.0	269 (1.15)	753 (1.25)	488 (1.47)	1510 (1.29)	<0.001
<−3.0 to −6.0	563 (2.40)	3277 (5.45)	2856 (8.63)	6696 (5.74)	
<−0.5 to −3.0	7295 (31.06)	22 226 (36.95)	14 475 (43.75)	43 996 (37.69)	
≥−0.5 to≤+0.5	10 167 (43.29)	26 455 (43.99)	12 878 (38.92)	49 500 (42.41)	
>0.5 to 2.0	3520 (14.99)	5284 (8.79)	1839 (5.56)	10 643 (9.12)	
>2.0	1670 (7.11)	2149 (3.57)	552 (1.67)	4371 (3.74)	
Median refractive error (D) (first and third quartiles)	−0.25 (−0.86, 0.25)	−0.50 (−1.25, 0.00)	−0.75 (−1.75, –0.25)	−0.5 (−1.25, 0.00)	<0.001
Range	−21.75 to 18.86	−21.75 to 16.0	−20.75 to 19.0	−21.75 to 19.0	
Median cylinder (D) (first and third quartiles)	−1.0 (−1.50, –0.50)	−0.75 (−1.25, –0.50)	−0.5 (−1.00, –0.50)	−0.75 (−1.25, –0.50)	<0.001
Range	−7.75 to 0.00	−7.75 to 0.00	−7.75 to 0.00	−7.75 to 0.00	

D, diopter; SE, spherical equivalent.

**Figure 1:**
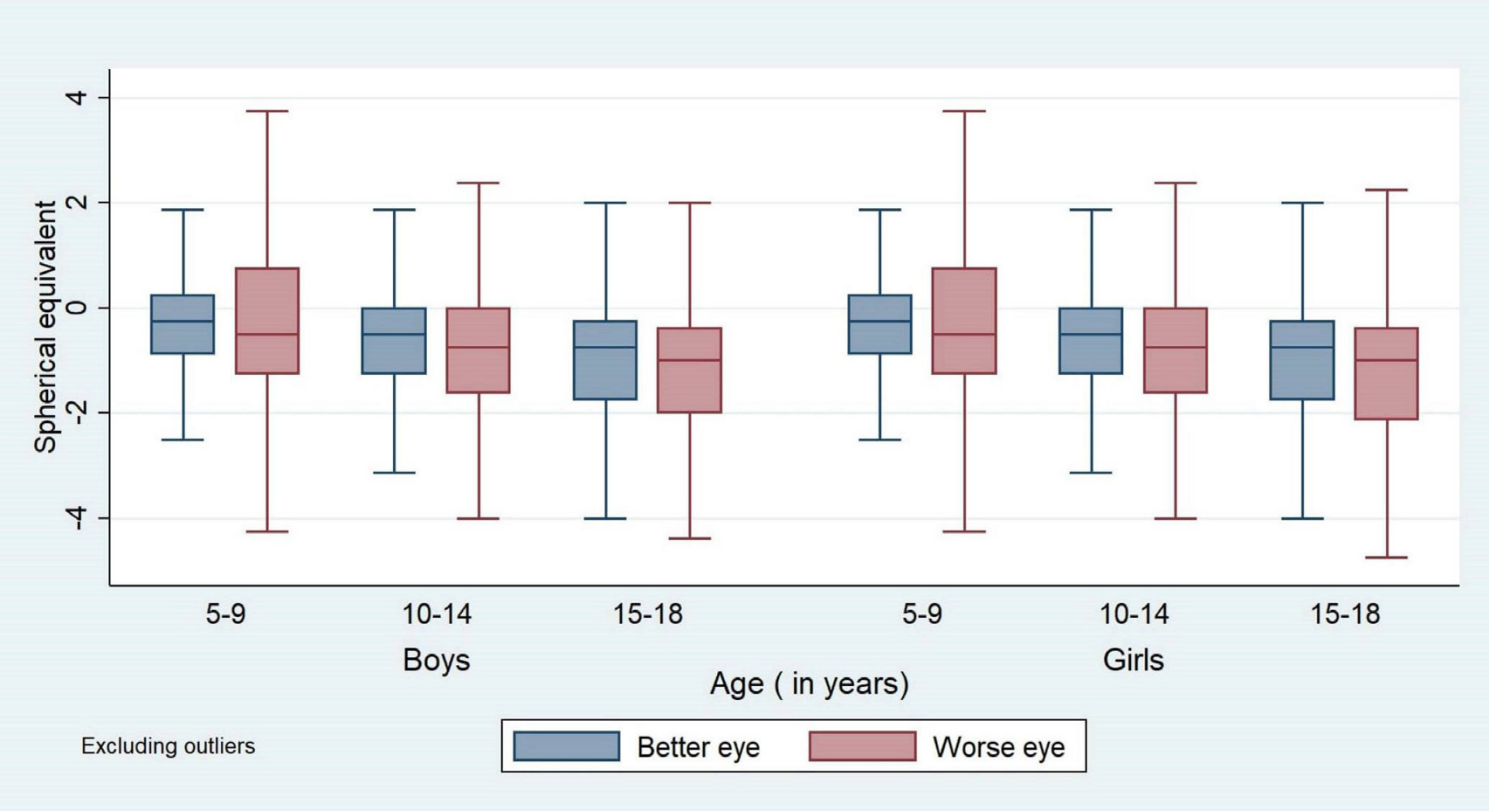
Distribution of spherical equivalent refractive error of each eye by age (years) and gender. Horizontal line markers show median values; error bars show IQR.

The mean ages of children participating in the primary screening and detailed evaluation were 11.5 years (SD ±3.3) and 12.2 years (SD ±3.1), respectively. The distributions of refractive errors in the better eye by age and gender are shown in figure 1. Table 1 shows the median SE refractive error in each eye in boys and girls. Median SE refractive error became progressively more myopic between ages 5 years and 9 years and between 15 years and 18 years among both boys and girls (p<0.001). In the whole cohort, the better eye median SE refractive error was −0.50 D (first and third quartiles −1.25 and 0.00 D) and median cylinder −0.75 D (first and third quartiles −1.25 and −0.50 D). The prevalence of SE myopia among the whole cohort was 3.00% and 3.55% in the better and worse eyes, respectively, while SE hyperopia prevalence was 0.53% in the better eye. Myopia prevalence based on spherical ametropia in the better eye was lower at 2.45%, while hyperopia was higher at 2.53%. This difference between SE and spherical ametropia is expected since refractive errors were expressed in negative cylinder format, and in the latter case, adjustment is not made for cylindrical values. For example, a myopic refractive error with a cylindrical component expressed as SE increases by half of the cylindrical value, but this increase is not made when expressed as spherical ametropia. Similarly, a hyperopic error is decreased when expressed as SE but not as spherical ametropia. These changes increase prevalence values for myopia and decrease them for hyperopia when SE is used.

Myopia was significantly higher among children in urban than rural locations at all age groups (p<0.001). Astigmatism was also higher in urban locations for children aged 5–14 (p<0.001) but not in those aged 15–18 (p=0.173). Low prevalence of anisometropia was found (table 2).

**Table 2:** P of each category of refractive error (better eye) at primary screening and detailed evaluation

Primary screening	Boys		Girls		Total	
Refractive error (D)	n=1 021 715	P (95% CI)	n=1 002 338	P (95% CI)	N=2 024 053	P (95% CI)
Mean SE						
Myopia (≤−0.5)	28 292	2.77 (2.74 to 2.80)	32 435	3.24 (3.20 to 3.27)	60 727	3.00 (2.98 to 3.02)
Hyperopia (≥+1.0)	5479	0.54 (0.52 to 0.55)	5176	0.52 (0.50 to 0.53)	10 655	0.53 (0.52 to 0.54)
Astigmatism (≥−0.5)	34 019	3.33 (3.29 to 3.36)	36 792	3.67 (3.63 to 3.71)	70 811	3.50 (3.47 to 3.52)
Anisometropia (SE_RE-SE_LE >1.0)	2733	0.27 (0.26 to 0.28)	2788	0.28 (0.27 to 0.29)	5511	0.27 (0.27 to 0.28)

Detailed evaluation	Boys		Girls		Total	
Refractive error (D)	n=65 043	P (95% CI)	n=72 105	P (95% CI)	n=137 148	P (95% CI)
Mean SE						
Myopia (≤−0.5)	28 292	43.50 (43.12 to 43.88)	32 435	44.98 (44.62 to 45.35)	60 727	44.28 (44.02 to 44.54)
Hyperopia (≥+1.0)	5479	8.42 (8.21 to 8.64)	5176	7.18 (6.99 to 7.37)	10 655	7.77 (7.63 to 7.91)
Astigmatism (≥−0.5)	34 019	52.30 (51.92 to 52.69)	36 792	51.03 (50.66 to 51.39)	70 811	51.63 (51.37 to 51.90)
Anisometropia (SE_RE-SE_LE >1.0)	2733	4.20 (4.05 to 4.36)	2788	3.85 (3.71 to 4.00)	5511	4.02 (3.91 to 4.12)

Spherical ametropia	Boys		Girls		Total	
Primary screening	n=1 021 715	P (95% CI)	n=1 002 338	P (95% CI)	N=2 024 053	P (95% CI)
Spherical ametropia						
Myopia (spherical value ≤−0.50)	21 788	2.13 (2.10 to 2.16)	25 921	2.59 (2.56 to 2.62)	47 709	2.36 (2.34 to 2.38)
Hyperopia (spherical value ≥+1.0)	22 399	2.19 (2.16 to 2.22)	23 036	2.30 (2.27 to 2.33)	45 435	2.24 (2.22 to 2.27)
Total	44 187	4.32 (4.29 to 4.36)	48 957	4.88 (4.84 to 4.93)	93 144	4.60 (4.57 to 4.63)
Astigmatism						
Any myopic astigmatism (cylinder value ≥−0.5)	34 019	3.33 (3.29 to 3.36)	36 792	3.67 (3.63 to 3.71)	70 811	3.50 (3.47 to 3.52)
Simple myopic astigmatism (‘0’ spherical value with any cylinder)	10 424	1.02 (1.00 to 1.04)	11 098	1.11 (1.09 to 1.13)	21 522	1.06 (1.05 to 1.08)
Compound myopic astigmatism (‘−’ spherical value with any cylinder)	14 812	1.45 (1.43 to 1.47)	17 022	1.70 (1.67 to 1.72)	31 834	1.57 (1.56 to 1.59)
Mixed astigmatism (‘+’ spherical value with any cylinder)	13 981	1.37 (1.35 to 1.39)	14 975	1.49 (1.47 to 1.52)	28 956	1.43 (1.41 to 1.45)

D, diopter; P, prevalence; SE, spherical equivalent.

Table 3 shows the prevalence of overall refractive error (myopia and hyperopia, better eye) in rural and urban locations in each state.

**Table 3:** P of refractive error (better eye) by gender, state and location at primary screening

State	Boys			Girls			Total		
	Children attending (n) n=1 021 715	n=33 771	P (95% CI)	Children attending (n) n=1 002 338	n=37 611	P (95% CI)	Children attending (n) N=2 024 053	n=71 382	P (95% CI)
Kerala									
Rural	7475	444	5.94 (5.41 to 6.50)	5771	361	6.26 (5.64 to 6.91)	13 246	805	6.08 (5.68 to 6.50)
Semirural	84 532	4907	5.80 (5.65 to 5.96)	79 725	5168	6.48 (6.31 to 6.66)	164 257	10 075	6.13 (6.02 to 6.25)
Semiurban	41 959	2345	5.59 (5.37 to 5.81)	45 325	2687	5.93 (5.71 to 6.15)	87 284	5032	5.77 (5.61 to 5.92)
Total	133 966	7696	5.74 (5.62 to 5.87)	130 821	8216	6.28 (6.15 to 6.41)	264 787	15 912	6.01 (5.92 to 6.10)
Madhya Pradesh									
Rural	75 752	2278	3.01 (2.89 to 3.13)	67 861	2095	3.09 (2.96 to 3.22)	143 613	4373	3.04 (2.96 to 3.14)
Semirural	49 550	1000	2.02 (1.90 to 2.15)	48 967	979	2.00 (1.88 to 2.13)	98 517	1979	2.01 (1.92 to 2.1)
Semiurban	81 375	2995	3.68 (3.55 to 3.81)	61 589	2422	3.93 (3.78 to 4.09)	142 964	5417	3.79 (3.69 to 3.89)
Total	206 677	6273	3.04 (2.96 to 3.11)	178 417	5496	3.08 (3.00 to 3.16)	385 094	11 769	3.06 (3.0 to 3.11)
Maharashtra									
Rural	82 873	1361	1.64 (1.56 to 1.73)	75 245	1601	2.13 (2.03 to 2.23)	158 118	2962	1.87 (1.81 to 1.94)
Semirural	45 811	924	2.02 (1.89 to 2.15)	40 940	1146	2.80 (2.64 to 2.96)	86 751	2070	2.39 (2.29 to 2.49)
Semiurban	53 644	1419	2.65 (2.51 to 2.78)	48 441	1846	3.81 (3.64 to 3.99)	102 085	3265	3.2 (3.09 to 3.31)
Total	182 328	3704	2.03 (1.97 to 2.10)	164 626	4593	2.79 (2.71 to 2.87)	346 954	8297	2.39 (2.34 to 2.44)
Tamil Nadu*									
Rural	73 496	2186	2.97 (2.85 to 3.10)	74 792	2523	3.37 (3.25 to 3.51)	148 288	4709	3.18 (3.09 to 3.27)
Semirural	96 228	2530	2.63 (2.53 to 2.73)	93 677	2933	3.13 (3.02 to 3.24)	189 905	5463	2.88 (2.80 to 2.95)
Semiurban and urban	159 207	9578	6.02 (5.90 to 6.13)	160 405	11 766	7.34 (7.21 to 7.46)	319 612	21 344	6.68 (6.59 to 6.77)
Total	328 931	14 294	4.35 (4.28 to 4.42)	328 874	17 222	5.24 (5.16 to 5.31)	657 805	31 516	4.79 (4.74 to 4.84)
West Bengal									
Rural	42 682	467	1.09 (1.0 to 1.20)	47 802	517	1.08 (0.99 to 1.18)	90 484	984	1.09 (1.02 to 1.16)
Semirural	68 905	401	0.58 (0.53 to 0.64)	84 878	571	0.67 (0.62 to 0.73)	153 783	972	0.63 (0.59 to 0.67)
Semiurban	58 226	936	1.61 (1.51 to 1.71)	66 920	996	1.49 (1.4 to 1.58)	125 146	1932	1.54 (1.48 to 1.61)
Total	169 813	1804	1.06 (1.01 to 1.11)	199 600	2084	1.04 (1.0 to 1.09)	369 413	3888	1.05 (1.02 to 1.09)

*Only in the state of Tamil Nadu four schools from urban areas were included.

P, prevalence.

In the univariate regression model (table 4), myopia in either eye was associated with female gender, increasing age, urban location, private school attendance and state (p<0.01). Multivariate regression confirmed that myopia was significantly more likely to occur in children aged 10–14 and 15–18 than in those aged 4–9 years, in girls, in private compared with government schools and in more densely populated areas (p<0.01). It also shows that compared with the state of Kerala, the risk of myopia was significantly higher in Tamil Nadu (p<0.01) but significantly lower in Madhya Pradesh, Maharashtra and West Bengal (p<0.01). This appears to be driven at least in part by differences in adult literacy rates between states,^20 21^ as illustrated in the multivariate model (table 4).

**Table 4:** Results of logistic regression analysis showing factors associated with myopia in the better eye

Reference variables	Categories	Univariate logistic regression			Multivariate logistic regression		
		OR	95% CI	P value	OR	95% CI	P value
Gender (boys)	Girls	1.17	1.16 to 1.19	<0.001	1.23	1.21 to 1.25	<0.001
Age 5–9 years	10–14	2.03	1.98 to 2.07	<0.001	2.16	2.11 to 2.21	<0.001
	15–18	3.16	3.09 to 3.24	<0.001	3.03	2.96 to 3.11	<0.001
Government-funded school	Private school	2.44	2.39 to 2.48	<0.001	2.21	2.17 to 2.26	<0.001
Kerala state (adult literacy* (%): overall 94.0, female 92.1, male 96.1)	Madhya Pradesh (adult literacy* (%): overall 69.3, female 59.2, male 78.7)	0.54	0.53 to 0.56	<0.001	0.67	0.65 to 0.69	<0.001
	Maharashtra (adult literacy* (%): overall 82.3, female 75.9, male 89.5)	0.59	0.57 to 0.61	<0.001	0.69	0.66 to 0.71	<0.001
	Tamil Nadu (adult literacy* (%): overall 80.1, female 73.4, male 86.8)	1.24	1.21 to 1.26	<0.001	1.58	1.54 to 1.62	<0.001
	West Bengal (adult literacy* (%): overall 76.3, female 70.5, male 81.7)	0.26	0.25 to 0.27	<0.001	0.48	0.46 to 0.50	<0.001
Location (rural)	Semirural	1.06	1.03 to 1.08	<0.001	0.97	0.94 to 0.99	=0.015
	Semiurban	2.01	1.96 to 2.05	<0.001	1.37	1.34 to 1.41	<0.001
	Urban	4.28	3.85 to 4.77	<0.001	2.51	2.25 to 2.79	<0.001

*Literacy rate is shown for each state (source: 2021 Census of India^21^).

## Discussion

The prevalence and progression of myopia in childhood have been widely discussed and is an area of intense research interest based on its significant health impact including vision impairment in uncorrected myopia and ocular pathology related to high myopia.^22^ Prevalence data depend in part on the definition of myopia, on geographical location (such as rural vs urban) and the refraction method (cycloplegia controlling accommodation-induced myopia). In the present study, myopia was defined in two ways, as SE and as spherical ametropia, the criterion for each being at least −0.50 D. Using a definition of SE at least −0.75 D and a similar age range (5–16 years), a recent study among schoolchildren in Tamil Nadu^23^ found myopia prevalence of 12%. This is five times higher than spherical ametropia prevalence in the present study. One possible explanation for this difference is that myopia prevalence is higher in Tamil Nadu than in other states. We found that the risk of myopia was indeed significantly higher in Tamil Nadu and Kerala than in the other states included here.

Few previous studies have reported on prevalence of refractive error among school children in Kerala. A 2018 study of newly prescribed refractive error (not including unchanged existing spectacles) in a population of Kerala school children aged 6–17 years found myopic astigmatism in 68.3%, simple myopia in 13.8%, hyperopic astigmatism in 13.1% and simple hyperopia in 1.20%.^24^ These numbers suggest that almost all of the children had newly prescribed refractive errors and are much higher than the 6.01% prevalence of overall refractive error found in Kerala in the present study. However, no criteria for refractive error were reported, so very low levels may have been included, and this may explain the very high prevalence.

Another study reported that 44% of a sample of Kerala school children aged 10–15 yeas had myopia.^25^ However, refractive error was not measured and the criterion for myopia was distance visual acuity poorer than Snellen 6/6. The reduced visual acuity may have been caused by other ocular factors such as high hyperopia, astigmatism or pathology, or non-ocular factors such as lighting or poor understanding of the acuity task. Previous studies in Kerala therefore do not provide a clear indication of refractive error prevalence in this state and are not comparable with the present findings.

A study on the prevalence of refractive error in school children aged 6–15 years in Maharashtra used more stringent criteria for myopia and hyperopia (≤−0.75 and >+2.00, respectively) than those of the present study.^26^ Myopia prevalence values of 3.16% and 1.45% were found in urban and rural areas, respectively, and hyperopia prevalence of 1.06% and 0.39% respectively. In the present study, overall prevalence (myopia and hyperopia combined) is 3.2% in semiurban areas and 1.87%–2.39% in rural to semirural areas of Maharashtra (table 3), broadly comparable to the previous findings. To our knowledge, no previous studies have reported on prevalence of refractive error among school children in West Bengal or Madhya Pradesh. Thus, there is little to no existing data on prevalence within or between the states that are included so far in the REACH programme. Based on 2011 census population data,^19^ the numbers of children screened represent about 3.3%, 1.6%, 1.1%, 3.7% and 1.4% of children aged 5–18 years in Kerala, Madhya Pradesh, Maharashtra, Tamil Nadu and West Bengal, respectively.

In the present study, the higher risk of myopia in Tamil Nadu and Kerala than in the other states may be related to a number of factors. First, about half (51%) of the cohort in Tamil Nadu attended schools located in rural or semirural areas, while about two-thirds were in these locations in the other states (63% in Madhya Pradesh, 66% in West Bengal, 67% in Kerala and 71% in Maharashtra; calculated from data shown in table 3). The high risk of myopia for children in Tamil Nadu may therefore be influenced by the fact that fewer of the children in that state were in rural environments.^27^ However, the high risk of myopia in Kerala cannot be explained in the same way. Kerala has the highest literacy rate in India (94%), while rates in the other states included here are lower (table 4).^28^ For women, the same ranking applies, and this is relevant because maternal education level is associated with children’s health and academic outcomes, and academic activities are linked with myopia. A study of mothers and their children in villages in Pakistan found that children whose mothers had some education spent significantly more time on study than those whose mothers were not educated.^29^ Since time spent indoors and on near work is a risk factor for myopia in children, higher levels of maternal literacy in Kerala may account for the relatively high prevalence of myopia there. These findings have implications for future patterns of refractive error prevalence across India, as adult literacy increases across the country^28^ suggest childhood myopia rates may be expected to grow further.

Previous studies have reported prevalence among school children aged 5–15 years in different locations in India. In Delhi and Gurugram (Haryana state), prevalence of 13.1% and 21.1% has been reported, respectively, with myopia defined as SE >−0.50 D.^30 31^ Another study of children aged 5–15 in Odisha state with the same definition for myopia found prevalence of 4.9%, closer to that of the present study^32^ perhaps due in part to the fact that the higher prevalence data were gathered using cycloplegia, while the latter study used cycloplegia only ‘as appropriate’.^32^ A systematic review of refractive error prevalence in children under 15 years of age in India^4^ included studies conducted in seven states. It showed an overall prevalence of 5.3% and values from 3% (Andhra Pradesh) to 7% (New Delhi) in population-based studies and from 2% (Maharashtra)^26^ to 14% (Gujarat) in school-based studies.

A meta-analysis of myopia prevalence in Indian school children^33^ showed the well-established higher prevalence in urban than rural locations but found four times higher increases in prevalence over a decade in rural children than in urban children. The authors suggested this reduced urban–rural gap reflects a changing environment in rural locations with increased availability of digital devices encouraging near work and time spent indoors.

A systematic review on the global prevalence of childhood myopia^34^ found that in South Asia (including India), prevalence ranged from 5.3% at 5 years to 13.9% at 18 years, reflecting the well-known increase in prevalence with age during childhood. They also found that South Asians living elsewhere (Australia, England and Singapore) were five times more likely to be myopic than those living in India or Nepal. This finding highlights the influence of social, cultural and environmental factors on myopia development, such as use of digital devices and indoor activities.^11 35^

In addition to prevalence, the severity of refractive error is important since this determines the likelihood of visual impairment and pathology. In the present study, the overall median SE refractive error was −0.50 D, higher than the level of −0.18 D previously found in children aged 5–16 years in Tamil Nadu.^23^ In contrast to these overall myopic values, a population-based study of urban school children in New Delhi^13^ found SE refractive error of +0.77 D in children aged 5–15 years. In that study, prevalence of myopia (defined as ≤−0.50, determined by cycloplegic retinoscopy) varied from 4.68% at 5 years to 10.8% at 15 years. They reported mean SE of 0.04 D at 5–10 years, −0.20 D at 11–12 years, and −0.50 D at 13–16 years. Comparable data are available in the earliest of the age groups in the present study with mean SE −0.41 at age 5–9. Relatively high myopia severity in the present study is likely to reflect, at least in part, the increased prevalence of myopia among Indian school children over recent decades.^33^

Studies of refractive error prevalence in school children have focused largely on myopia, for important reasons related to retinal health and impact on function. However, hyperopia, with potential negative impact on clear, single vision and potentially on literacy^6^ should also be considered. The overall prevalence of hyperopia in the present study was only 0.53% when defined as SE but increased to 2.53% using spherical ametropia without cylindrical power adjustment. As explained earlier, most previous studies on refractive error prevalence in children use SE and would be expected to find relatively low prevalence of hyperopia because the positive hyperopic error is reduced by half of the negative cylinder. The use of both approaches shows that, depending on levels of astigmatism in the population, the prevalence and severity of myopia are higher and those of hyperopia are lower when SE is used than when spherical ametropia is used. This raises questions about the use of SE for refractive error prevalence and severity estimates and indicates a need to consider astigmatism levels when interpreting SE data on myopia and hyperopia prevalence.

### Strengths and limitations

This study has several strengths, including its size and wide scope. Due to the large-scale school vision screening of the REACH programme, this study included data from more than 2 million children in over 10 000 schools at multiple locations across India. The data were collected using uniform protocols across all schools, ensuring comparability between locations and school categories. High rates of participation were also achieved.

We have reported prevalence based on both the better and worse eyes. This approach illustrates the extent to which prevalence may be affected by reporting only one of these values and allows fairer comparison with previous research based on either of these values.

This study provides the first state-specific data on prevalence of refractive error among school children in the states of Madhya Pradesh and West Bengal. New data are also provided on prevalence in Kerala, Tamil Nadu and Maharashtra, where very little data were previously available.

In contrast to previous studies of this kind in India, in which screening has been conducted in open environments with daylight illumination, the present study used a classroom environment with ambient lighting. This replicates the environment usually experienced by the children while learning and may have enabled a realistic assessment of vision as experienced by the child.

Refractive error was assessed in most children without cycloplegia, and this may have affected the prevalence estimate. The Refractive Error Study in Children^36^ protocol includes cycloplegic refraction to exclude the possibility of myopia being artificially increased or hyperopia decreased by accommodation. In the present study, non-cycloplegic refraction may have led to a moderate myopic shift, especially in younger children,^37^ and a higher myopia prevalence than might be expected if cycloplegia were used in all children. It is important to note, though, that the non-cycloplegic condition is the natural state, so our estimates reflect the children’s habitual levels of refractive error.

## Conclusions

Refractive error, especially myopia, is common among Indian school-going children, and comparisons with earlier studies suggest that it may be growing. This study is among the first to apply a common vision screening and refraction protocol across multiple states of India, and interstate differences appear to be driven at least in part by differences in adult literacy. This is of importance for programme planners: as literacy rates rise in India, further increases in childhood myopia, with attendant demands on healthcare resources, may be anticipated.

## Data Availability

All data relevant to the study are included in the article or uploaded as supplementary information.
